# Urology Training in sub-Saharan Africa: A Survey of Training Directors

**DOI:** 10.4314/ejhs.v34i5.8

**Published:** 2024-09

**Authors:** Anteneh Tadesse Kifle, Saleh Abdelkerim Nedjim, Ziba Ouima Justin Dieudonne, Kaleab Habtemichael Gebreselassie, Marcella Derboise Christelle Biyouma, Rachid Aboutaieb, Mahamat Ali Mahamat, Emmanuel Ugbede Oyibo, Nathnael Abera Woldehana, Chandra Shekhar Biyani

**Affiliations:** 1 Department of Surgery, PCEA CHOGORIA HOSPITAL, KENYA; 2 Department of Urology, Centre Hospitalier Universitaire La Renaissance, N'djamena, Chad; 3 Department of Urology, Centre Hospitalier Universitaire Yalgado Ouedraogo, Ouagadougou, Burkina Faso; 4 Department of Surgery, Urology Unit, Worabe Comprehensive Specalized Hospital, Worabe, Ethiopia; 5 Urology Unit, Department of Surgery, Douala Laquintinie Hospital, Douala, Cameroon; 6 Department of Urology, Centre Hospitalier Universitaire Ibn Rochd, Casablanca/Morocco; 7 Department of Urology, Hôpital Général de Référence Nationale, N'Djamena, Chad; 8 Department of Urology, Usmanu Danfodiyo University Teaching Hospital, Sokoto, Nigeria; 9 Department of Public Health, Johns Hopkins Bloomberg School of Public Health, USA; 10 Department of Urology, St James's University Hospital, Leeds Teaching Hospitals, UK; 11 Modern Urology for Africa

**Keywords:** Urology, surgical Training, Sub-Saharan Africa, Global surgery, Global Urology, endourology

## Abstract

**Background:**

Urology is one of the most rapidly evolving and technologically advanced surgical specialties. However, training programs in Sub-Saharan Africa (SSA) face unique challenges. This study aimed to assess the current status of urology training in SSA and identify areas for improvement, providing crucial insights into the strengths and limitations of these programs.

**Methods:**

A 24-discrete items online survey was developed with “Google Forms” in English and French. The questionnaire was composed of two parts. The first part assessed general information about the urology training, and the second part evaluated the consultant's or department head's ability to perform and teach endourology procedures.

**Result:**

A total of 25 responses were received from 18 countries. The oldest training center is the University of Cape Town, South Africa; the program started in 1950. The number of consultants in the department ranges from 1 to 12, with an average of 4.79. Twenty of the training sites have a compulsory general surgery clinical attachment in their program. Fourteen of the training sites stated that research is mandatory in their curriculum. Fourteen of the centers reported providing laparoscopic urology surgery. Cystoscopy is the most mastered procedure and percutaneous nephrolithotomy (PCNL) is the most difficult procedure to gain any experience with by residents.

**Conclusion:**

Urology training in sub-Saharan Africa started late but is increasing in number through time. Urology training in Africa faces multiple challenges, including inadequate number of experts, limited availability of advanced equipment and simulation-based training sites. Training institutions should be encouraged to facilitate research and basic urological skills training.

## Introduction

Global surgery is defined as an area of study, research, practice, and advocacy that seeks to improve health outcomes and achieve health equity for all people who need surgical and anesthesia care, with a special emphasis on underserved populations and populations in crisis (1). According to a Lancet Commission report, 5 billion people lack access to safe, affordable surgical and anesthesia care when needed, and most of these people reside in low- and middle-income countries (LMICs), according to the World Bank designation (2).

Although surgery has been increasingly recognized as a crucial component of universal health coverage, urology has not received proportional attention. Urology is one of the most rapidly evolving and technologically advanced surgical specialties. Urology has many subspecialties, including endourology, andrology, urological oncology, pediatric urology, reconstructive urology, laparoscopic and robotics. The technological advancements, including laparoscopy, endourology, and the recent significant advance in robotic surgeries, have made the stream a hub for the advancement of minimally invasive surgeries (3).

Urological surgical conditions diminish the quality of life and decrease productivity, thereby affecting the well-being of the individual and the community at large (1). The five most commonly encountered urologic conditions in SSA were benign prostatic hyperplasia, urethral stricture, prostate cancer, bladder cancer, and urethral or ureteral trauma (4). The burden of urologic disease in SSA is expected to increase as the ageing population, exposure to risk factors like smoking, and “patient-led” screening for health conditions like prostate cancer (5). Most of these urologic conditions are treated by general surgeons and general practitioners, who have limited urological training, as the number of urologists is limited significantly. In addition, most of the urologists reside in big cities, making accessibility to the general population troublesome. Most hospitals in SSA also lack basic endourologic equipment, which significantly affects the service delivery by urologists (6).

Previously, a general surgery specialty qualification was needed before undergoing a urology-specific fellowship in most African countries. The Urology Fellowship (Fellow of the College of Surgeons) training after passing a Member of the College of Surgeons (MCS) examination was started in 1999 by the College of Surgeons East Central and South Africa (COSECSA) (7). This was shortly followed by direct national urology residency programs (7–9). Despite this new approach, urology training in African countries is not yet fully structured. Compared to developed countries, at the end of the training, there is a difference in the technical skills of young urologists, depending on the centers. Training is still mainly based on the Halstedian model. In some parts of the developing world, such as most of SSA, training in modern urology is still carried out directly in the operating room on real patients (10). In developing countries, complex endo-urological and laparoscopic procedures are taught on models using simulators and under the supervision of a mentor (11). This reduces the learning curve and ensures greater patient safety during real-world procedures.

Compared to other regions of the world, the challenges of urological disease diagnosis and treatment are highest in SSA. The main difficulties are linked to low levels of infrastructure, funding, and resources. Added to this are the difficulties associated with quality of local training and the availability of qualified experts or specialists (7,12). In 2006, Olapade-Olaopa et al. reported specific issues affecting the practice of urology. These include the limited number of trained urologists, the limited availability of modern equipment and specific drugs, insufficient support or compensation from centers, and other epidemiological and cultural problems (13).

Sub-Saharan Africa (SSA) comprises the countries in Africa that do not have a Mediterranean coastline or are south of the Saharan desert. According to a 2023 World Bank report, the region is home to over 1.1 billion people, accounting for over 15% of the world population.

Although surgery has been increasingly recognized as a crucial component of universal health coverage, urology has not received proportional attention. Delivery of subspecialty surgical care in low-resource settings, such as SSA, faces numerous challenges, including limited access to specialized equipment and trained personnel (14). While general surgery training incorporates urology into its training (15,16) few studies are exploring the role of urology residency training. This study aims to fill this gap by assessing the current status of urology training programs in SSA and identifying areas for improvement

## Methods

An online survey was developed in Google Forms with 24 discrete items written in English and French. The survey was adaptive, and its length varied depending on the information provided by the respondent. The questionnaire was validated through pilot testing with a small group of urology training directors to ensure clarity and reliability. The presence of urology residency training in each Sub-Saharan country was searched on the internet and confirmed by communicating with urologists in each Sub-Saharan African country. Surveys were distributed to the department head or one of the consultants in each training institution in SSA.

The questionnaire was adapted from previous publications (17). The questionnaire was composed of two parts. The first part assessed general information about the urology training, including its curriculum, language of instruction, how long it has been giving the training, and how many residents have graduated and are currently enrolled in the program. The second part evaluated the consultant's or department head's ability to perform endourology procedures. Data were analyzed using descriptive statistics to summarize the key findings.

## Results

**Figure 1:**
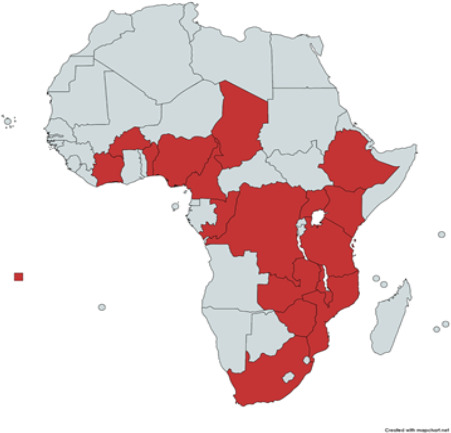
Countries with confirmed response and urology training

A total of 25 responses were received from 18 countries. The representatives of four training centers from Nigeria responded, followed by Senegal, Ethiopia, and Zimbabwe, each accounting for two centers. Urologists from two countries— Burundi and South Sudan—have confirmed the unavailability of urology training in their countries (*Figure 1*). By geographic region, the countries are distributed as follows: 1 from Southern Africa, 9 from East Africa, 3 from Central Africa, and 7 from West Africa.

For the survey as a whole, sixteen training programs began on average in 2010 and onward. The oldest training center is the University of Cape Town, South Africa; the program started in 1950. At the time of the survey, Chad had been running its training program for less than a year. *Table 1* shows all centers by country and training program start date.

**Table 1:** all centers by country and training program start date

East Africa		

Country	Training Center	Program starting year
**Ethiopia**	Addis Ababa University	2009
**Ethiopia**	St. Paul's Hospital Millennium Medical College	2017
**Kenya**	Defense Forces Memorial Hospital, Nairobi	2021
**Malawi**	Kamuzu central hospital, Lilongwe	2017
**Tanzania**	Kilimanjaro Christian Medical University College,	1999
**Uganda**	Mulago National Referral Hospital, Kampala	2013
**Zambia**	UTH University Teaching Hospital, Lusaka	2018
**Zimbabwe**	Harare Central Hospitals, Harare	1998
**Zimbabwe**	University of Zimbabwe	1996
**West Africa**		

**Country**	**Training Center**	**Program starting year**

**Benin**	CHU Hubert Koutoukou MAGA, Cotonou	2008
**Burkina Faso**	CHU Yalgado Ouedraogo, Ouagadougou	2016
**Ivory Coast**	CHU de Treichville, Abidjan	2016
**Mali**	CHU du Point G, Bamako	2009
**Nigeria**	Usmanu Danfodiyo University Teaching Hospital	2005
**Nigeria**	Lautech Teaching Hospital, Osogbo	2022
**Nigeria**	Defense Federal Medical Center, Keffi	2018
**Nigeria**	University of Abuja Teaching Hospital, Abuja	2010
**Senegal**	Hôpital Aristide Le Dantec, Dakar	1999
**Senegal**	Université Cheikh Anta Diop, Dakar	1980
**Sierra Leone**	University of Sierra Leone Teaching Hospitals	2018
**Central Africa**		

**Country**	**Training Center**	**Program starting year**

**Cameroon**	Hôpital Central de Yaoundé, Yaoundé	2010
**Chad**	CHU de Référence Nationale, N'djamena	2023
**Congo**	CHU de Brazzaville, Brazzaville	2021
**Southern Africa**		

**Country**	**Training Center**	**Program starting year**

**South Africa**	University of Cape Town	1950

The language of instruction reported as English in 14 centers, French in 8 centers, and Portuguese and bilingual (English and French) in one center each. The number of consultants in the department ranges from 1 to 12, with an average of 4.79 and a median of 5. 16 respondents responded that it takes 5 years to complete a urology residency, while 4 responded 4 years and 3 responded 6 years. In one training center, it takes 7 years to complete.

Twenty of the training sites have responded to have compulsory general surgery clinical attachment. The transition occurs in the first two years of the eight programs, the first three years of five programs, and the first year of four programs. In two of the programs, the general surgery attachment can be taken as an elective throughout their residency.

Fourteen of the training sites stated research is mandatory in their curriculum. Five programs are affiliated with training institutions or NGOs from high-income countries. Trainees from seven training programs are required to undertake external attachment outside of their country of training to acquire specific skills. The destinations for these external attachments are India and France by two respondents each, and Portugal, the USA, the UK, and Senegal each by one training site. Endourology skill training is the reason for six of the training sites, while one responded for uro-oncology and laparoscopy, sending their residents abroad for training.

For items defining sub-specialty practice, the participation rate (responses received) varied. Fourteen of the centers reported providing laparoscopic urology surgery. *Table 2* shows the different sub-specialties, and the percentage of procedures performed in all the centers surveyed. Fourteen (56%) respondents provide some type of laparoscopic procedures. Eleven respondents (44%) reported that they did not perform laparoscopic surgery at their center. Laparoscopic varicocelectomy and hernia repair are the most common laparoscopic procedures performed by those who responded.

**Table 2:** Urologic procedures performed stratified by subspecialty

Sub-specialties	Procedures	Procedures by center	
		Number (n/N)	Percent
Open surgery for calculus	Nephrolithotomy	21/25	84
	Pyelolithotomy	25/25	100
	Ureterolithotomy	20/25	80
	Bladder stone extraction	24/25	96

Endourology	Cystoscopy	25/25	100
	Endoscopic urethrotomy	25/25	100
	Transurethral prostate resection	24/25	96
	Transurethral bladder resection	23/25	92
	Ureteroscopy	12/25	48
	Percutaneous nephrolithotomy	13/25	52

Laparoscopy	Varicocele	11/14	78.6
	Hernia	10/14	71.4
	Genital prolapse	6/14	42.9
	Vesico-vaginal fistula	4/14	28.6
	Pyeloureteral junction syndrome	9/14	64.3
	Nephrectomy	11/14	78.5

Oncology	Penectomy	20/25	80
	Radical orchiectomy	25/25	100
	Radical prostatectomy	18/25	72
	Radical cystectomy	19/25	76
	radical nephroureterectomy	20/25	80
	radical nephrectomy	22/25	88
	Adrenal surgery	18/25	72
	Retroperitoneal Lymph Node Dissection	8/25	32

Reconstructive And functional urology	Pyeloplasty	25/25	100
	Urethroplasty	25/25	100
	Neobladder creation	16/25	64
	Bladder Augmentation	18/25	72
	Penile implant	6/25	24

Andrology	Testicular Biopsy	17/25	68
	Transurethral resection of Ejaculatory ducts	11/25	44
	Vasogram and surgery of the vas deferens and epididymis	5/25	25

Pediatric urology	Hypospadias repair	25/25	100
	Bladder exstrophy and epispadias repair	17/25	68
	Orchidopexy	24/25	92
	PUV ablation	15/25	60
	Ureteric surgeries, including pyeloplasty and reimplantations	21/25	84

**Figure 2:**
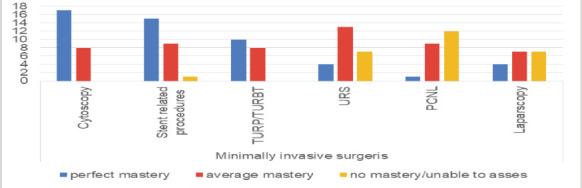
Level of trainees after completion of training

The training directors gave their opinion on trainees' autonomy and satisfaction at the end of training in 24 cases for cystoscopy, double J stent placement, ablation, and ureteroscopy. In 22 and 19 cases, they gave their opinions on PCNL and TURP, respectively. The level of confidence in endourological procedures by the residents varies from program to program. The level has been assessed on 3-point scale (perfect mastery, average mastery, or no mastery or procedure not practiced in the center) and is represented in the following figure (*Figure 2*).

A text box for free comments was left for the trainers to comment on advancing urology at the end of the questionnaire, some answered:

- Facilitating access to training and, above all, developing endourology
- The need to set up a regular exchange program between centers for skills transfer
- Equip centers with endourology equipment and make consumables available

## Discussion

**Urology training program in African and variability**: The first formal urology residency training was started at Johns Hopkins Hospital in Baltimore, Maryland, USA, in 1915. With contributions from many other centers and technological advancement, the types of training skills and knowledge to acquire have changed a lot over time. The oldest formal urology residency training centers in this study were South Africa (1950) from the south, Senegal (1980) from the west, and KCMC (2004). Since 1999, there has been a fellowship program developed by the College of Surgeons of East, Central, and Southern Africa (COSECSA), allowing general surgeons to undergo specific endourology training at KCMC (7). Sixteen (65%) responding centers started urology training after 2000. This clearly shows a major delay in starting the training program in urology, which has a direct influence on the development of the discipline on the continent. The beginning of surgical training in one's own country has been associated with a significant improvement in the number of experts and a decrease in the brain drain associated with training in high-income countries (6,8).

The surgical training in Africa almost always follows the Halstedian tradition of a defined apprenticeship where the trainee starts with participation, observation, graded participation, and responsibility sharing until he or she acquires the same ability as the trainer. To get the best outcome, training centers should have a team of experienced urologists in different subspecialty domains. 11/25 centers have less than 5 consultants. Only two centers have more than 12 consultants.

There is a difference in the number of years it takes to complete urology training across countries. The United Kingdom trains its residents for 9 years, New Zealand for 7 years, and the USA and Canada train for 5 years (17). In our study, 17 (68%) centers train for 5 years, while 4 centers train for 4 years. The six-year training programme at the West African College of Surgeons' affiliated centers consists of three years of general surgery instruction and three years of urology training. A four-year urology curriculum has also been developed in the USA. It has shown improved performance on a national cognitive test and high satisfaction with the program among educators and residents (18).

Urology research has focused on the study of urological disease, urological techniques, procedures, and technologies to improve the safety, effectiveness, and efficiency of treatments. Africa lags in enjoying these benefits, primarily because of a lack of human resources, infrastructure, and funding (19). Fourteen training programs require research as a mandatory part of their curriculum. This has to be encouraged to be adopted by all training programs. This will expose the trainees to evidence-based medicine and encourage innovations.

**Challenges in equipment and resources**: In high-income countries, urology training programs have incorporated simulation-based training into their curriculum, thereby evaluating the skills of the trainee objectively and consistently. This has improved patient safety and efficient skill transfer based on the trainees' pace. This model allows for safe and efficient training curricula with the ability to evaluate skills objectively and consistently (20,21). In the UK, a 5-day intensive simulation-based urology boot camp gives real-time feedback to the trainee by their trainer, and also guiding their progress (22). Despite the financial, logistical, and behavioral challenges of integrating simulation-based training into the curriculum, similar results have been observed in many SSA countries like Uganda, Rwanda, Tanzania, and Kenya (21).

Urological training in developing and low- and middle-income countries is mostly unstructured, with significant variation in the standards of graduates from various programs (10). This has been demonstrated in our study as well, 50% and 29% of the training sites are not practicing PCNL and ureteroscopy (URS), respectively, while 44% do not have laparoscopic urology services. thereby unable to train the residents and assess their capability in performing the procedure, while 50% and 70% train the residents to the level of mastery average or more in PCNL and URS. Some of the reasons stipulated for the unavailability of upper tract endoscopic procedures are ascribed to equipment, including consumables, and the required skill gap (23). There is also a gap in the provision of infertility investigation and treatment, as well as in penile prosthesis implantation.

Urology remains one of the rapidly evolving surgical specialties, and there is great variation between training programs in different countries (24). Urology training is subject to many theoretical, practical, and scientific requirements. Validation of these requirements assumes that graduate trainees have acquired the knowledge necessary to practice urological surgery (25). An analysis of Figure 2 shows, in order, high resident satisfaction with cystoscopy skills (66.7%), double-J catheter procedures (53.8%), ureteroscopy (54.2%), and transurethral resection of the prostate (42.1%). Satisfaction with competence in percutaneous nephrolithotomy was as low (4.50%). This represents the average for centers responding to the survey. In isolation, there is considerable variation between centers. The two reasons stipulated for these are the unavailability of equipment and the lack of qualified personnel for endourological procedures. In addition, the learning curve depends on the type of procedure.

The learning curve in surgery is the number of cases required to perform a procedure solo with a reasonable operative time and acceptable complication rate, resulting in an adequate postoperative clinical outcome and a shorter hospital stay (26). Regarding the learning curve for flexible cystoscopy, Berrosteguieta et al. noted perfect achievement of the technique after performing 6 to 10 procedures (27). Percutaneous nephrolithotomy is a difficult procedure with a long learning curve (28). According to Allen et al., it takes up to 115 procedures to achieve excellence (29). According to the data collected, laparoscopic surgery is performed in 61.1% of centers. In the majority of cases (38.9%), the skill acquired at the end of training is open laparoscopy. Laparoscopic surgery in urology differs from its counterparts in general surgery in that there are no relatively simple procedures. It has traditionally been regarded as a sub-specialized procedure (30).

The variability in operative case volumes across different training centers highlights the need for standardizing training requirements to ensure consistent competency among graduates (16). Additionally, innovative approaches, such as a novel online, modular, flipped-classroom curriculum, have shown promise in improving surgical education and addressing resource constraints (31). Surgical simulation has also been shown to significantly improve training outcomes, providing a safe and effective platform for skill acquisition (32). In a survey of 21 reference centers in Africa, Modern Urology for Africa reported that 28.5% have a simulation unit (33). All should evaluate and adjust the simulation center's capabilities, technological advancements, and curricula. Because urological training needs to integrate educational technologies for better and faster skill acquisition, it reduces the learning curve, builds trainee confidence, and enables optimal patient care (20).

**Recommendations for improvement**: In their report published in Lancet Global Health in 2015, Mera et al. have identified the density of specialized health personnel as a health indicator (34). The World Bank, in turn, has identified the density of specialized health personnel as a development indicator (35). In 2006, Olapade-Olaopa et al. noted the shortage of surgeons in Africa. This shortage has particularly affected urology (13). As far back as 1996, an inadequacy in the quality and quantity of surgical services available was noted in several African countries. On top of this, access for African doctors to specialization programs outside the continent was becoming increasingly difficult. This led some countries to create the College of Surgeons of Eastern, Central, and Southern Africa (COSECSA). The main aim of this college was to train surgeons in Africa by setting up a network between member countries, enabling them to share resources (36). At the same time, international partnerships between high, low- and middle-income countries continue in a sporadic and disparate way to train surgical personnel. These partnerships take the form of visits by an expert to resource-constrained centers or the dispatch of a surgeon from an international reference center. Both types of partnerships have raised several concerns (37). This survey clearly shows the importance of internships outside of Africa. This internship aims to enable African surgeons to acquire new skills. In this study, the main skills sought are the acquisition of endourology techniques, followed by laparoscopic and oncologic urology. This type of partnership has advantages but also poses some problems that need to be brainstormed: expatriation, financial problems, and learning in environments with other realities that cannot be produced back in Africa. Added to this is the risk of brain drain. Bentounsi et al. reiterated the need to design locally based surgical training programs with a real and lasting impact (37).

Organizing regular on site workshops based on local target needs can facilitate the acquisition of skills by an entire local group. This type of partnership needs to be further developed, especially between African centers. This can help strengthen collaboration and easily evaluate shared knowledge. Urolink has equipped centers in KCMC and Hawassa where the local urologists were taught by visiting consultants on different aspects of endourology and reconstructive surgeries (21). Other international groups, like IVUmed, Société Internationale d'Urologie, and the European Urology Association, with their philanthropic committees, and the Intensive Interactive Training Program (IITP) by Dr. Jacques Bogdanowicz, are also contributing a lot to the skill transfer by onsite workshops (6).

Overall, to improve urology training in sub-Saharan Africa, the following points should be considered: the creation of programs in all countries; identification of reference centers for sub-specialties; networking between African programs; organization of workshops; and mobility to acquire specific skills with a long learning curve. Future research should focus on developing standardized training requirements and exploring innovative educational approaches to address these challenges (31).

There are several limitations to this study, although it provides data relevant to urological training in Sub-Saharan Africa, certain urology training institutions, including as University of Rwanda, Windhoek Central Hospital, and Kenyatta University, have not responded to us. The questioner is also dependent on the perception of the training director, which may also influence the result of our study. The answer on the provision of the urologic procedures was not confirmed by an independent investigator or the researchers. We acknowledge these drawbacks and recommend future researchers to include these factors in their study.

In conclusion, although urology training in SSA commenced later than in other regions, it has shown notable progress. The number of training centers and acceptance capacity of training centers from COSECSA, WACS, and national programs is rising. However, disparities persist, particularly in access to advanced surgical techniques and equipment. Strategic investments and international collaborations are crucial to further enhance training quality and healthcare outcomes. Cooperation from high-income countries and institutions based on local needs and on site has shown a clear success. Future research should focus on standardizing training requirements and finding novel educational and partnership approaches.
